# Construction of machine learning models based on transrectal ultrasound combined with contrast-enhanced ultrasound to predict preoperative regional lymph node metastasis of rectal cancer

**DOI:** 10.1016/j.heliyon.2024.e26433

**Published:** 2024-02-15

**Authors:** Xuanzhang Huang, Zhendong Yang, Wanyue Qin, Xigui Li, Shitao Su, Jianyuan Huang

**Affiliations:** a Department of Ultrasound, The First Affiliated Hospital of Guangxi Medical University, Nanning, PR China; b Department of Radiation Oncology, The First Affiliated Hospital of Guangxi Medical University, Nanning, PR China

**Keywords:** Rectal cancer, Regional lymph node metastasis, Transrectal ultrasound, Contrast-enhanced ultrasound, Machine learning, Predictive model

## Abstract

**Purpose:**

Constructing a machine learning model based on transrectal ultrasound (TRUS) combined with contrast-enhanced ultrasound (CEUS) to predict preoperative regional lymph node metastasis (RLNM) of rectal cancer and provide new references for decision-making.

**Materials and methods:**

233 patients with rectal cancer were enrolled and underwent TRUS and CEUS prior to surgery. Clinicopathological and ultrasound data were collected to analyze the correlation of RLNM status, clinical features and ultrasound parameters. A 75% training set and 25% test set were utilized to construct seven machine learning algorithms. The DeLong test was used to assess the model's diagnostic performance, then chose the best one to predict RLNM of rectal cancer.

**Results:**

The diagnostic performance was most dependent on the following: MMT difference (36), length (30), location (29), AUC ratio (27), and PI ratio (24). The prediction accuracy, sensitivity, specificity, precision, and F1 score range of KNN, Bayes, MLP, LR, SVM, RF, and LightGBM were (0.553–0.857), (0.000–0.935), (0.600–1.000), (0.557–0.952), and (0.617–0.852), respectively. The LightGBM model exhibited the optimal accuracy (0.857) and F1 score (0.852). The AUC for machine learning analytics were (0.517–0.941, 95% CI: 0.380–0.986). The LightGBM model exhibited the highest AUC (0.941, 95% CI: 0.843–0.986), though no statistic significant showed in comparison with the SVM, LR, RF, and MLP models (*P* > 0.05), it was significantly higher than that of the KNN and Bayes models (*P* < 0.05).

**Conclusion:**

The LightGBM machine learning model based on TRUS combined with CEUS may help predict RLNM prior to surgery and provide new references for clinical treatment in rectal cancer.

## 1 Introduction

Colorectal cancer is a common malignancy of the digestive tract. According to Global Cancer Statistics 2020 (GLOBOCAN 2020), colorectal carcinoma is one of the top five causes of cancer-related mortality, accounting for 10% of new cancers worldwide [1]. The status of regional lymph node metastasis (RLNM) directly affects the formulation of preoperative treatment plans for rectal cancer. For patients with early rectal cancer without RLNM (cT1N0M0), methods such as local endoscopic resection can be used to avoid overtreatment. Locally advanced rectal cancer treatment is mainly treated with neoadjuvant chemoradiation, followed by total mesorectal excision (TME). At the same time, RLNM plays a pivotal role in postoperative tumor recurrence and distant metastasis [2]. Therefore, improved accuracy in clinical lymph node status at diagnosis is critical in tumor staging, treatment options and prognosis for rectal cancer. However, there is no reliable evaluation model thus far. Meta-analysis results [3] showed that the sensitivity and specificity of the three imaging methods of endoscopic ultrasound (EUS), computed tomography (CT), and magnetic resonance imaging (MRI) in the assessment of lymph node involvement in rectal cancer were similar (EUS, 57 and 80%; CT, 79 and 76%; MRI, 77 and 76%). Current imaging studies are of limited predictive value for RLNM prior to rectal cancer surgery.

Although Machine Learning (ML) modeling has been more frequently used in medical imaging, there are still relatively few studies on the application of ML modeling to study ultrasonography in rectal cancer. To the best of our knowledge, the application of machine learning models combined with transrectal ultrasound imaging parameters to predict lymph node metastasis in the current study has not been reported so far, which is the contribution of this study. The existing diagnosis of lymph node metastasis in rectal cancer mostly relies on CT, MRI, and ultrasound for single or combined assessment. In this study, based on the original diagnostic methods, we not only combined clinical data, but also improved and upgraded the research methods, and applied deep learning methods to explore the relationship between microfluidic perfusion and lymph node metastasis of rectal cancer, and achieved better diagnostic efficacy. This is also a kind of exploratory research.

Neoangiogenesis in malignant tumors is closely related to tumorigenesis and developmental processes. Angiogenesis provides oxygen to the tumor, excretes metabolic waste, and supplies many branched blood vessels, which is essential for tumor invasion and metastasis [4]. In recent years, with the development of ultrasonic medicine, contrast-enhanced ultrasound (CEUS) has become a reliable method to quantitatively study the process of tumor angiogenesis [5] However, there is little information regarding the application of microbubble contrast agents in intestinal diseases, especially the time-intensity curve (TIC) of transrectal ultrasound (TRUS) in the quantitative assessment of rectal tumor angiogenesis [6,7]. Therefore, this proposed research quantitatively assessed the microcirculatory perfusion status by ultrasonography to predict rectal cancer lymph node metastasis.

At present, most of the predictions of rectal cancer lymph node metastasis are made only from the visual level of the human eye to diagnose medical imaging data, and there are limitations such as smaller utilization of image information and more subjective judgment. Radiomics, proposed by Lambin in 2012, combines medical imaging, computer graphics and related data science, which is characterized by high throughput, multidimensionality, and big data [8]. From another point of view, it provides a new approach to the diagnosis of diseases. Compared to traditional medical data, these high-throughput data require more matching data processing methods to build better models, and as a branch of artificial intelligence in the era of big data, machine learning empowers machines to self-learn and recognize data in specific tasks. The study focuses on addressing the following issues：1. quantitatively analyze the correlation between rectal tumor neovascularization and lymph node metastasis based on transrectal ultrasound sonographic time-intensity curves (TICs); 2. establish seven machine learning models based on clinical-ultrasound imaging parameters; 3 compare the prediction accuracies of each model for lymph node status and screen the best prediction model.

## 2 Materials and methods

### 2.1 Enrollment and eligibility

A total of 260 rectal cancer patients diagnosed by histopathology were recruited from the First Affiliated Hospital of Guangxi Medical University between October 2018 and July 2021. The inclusion criteria were as follows: (1) The pathological results were proven to be rectal adenocarcinoma; (2) The number of lymph nodes involved in lymphadenectomy was larger than 12; (3) No chemotherapy or radiotherapy was performed prior to surgery; (4) The clinical data was complete.

**Fig. 1:**
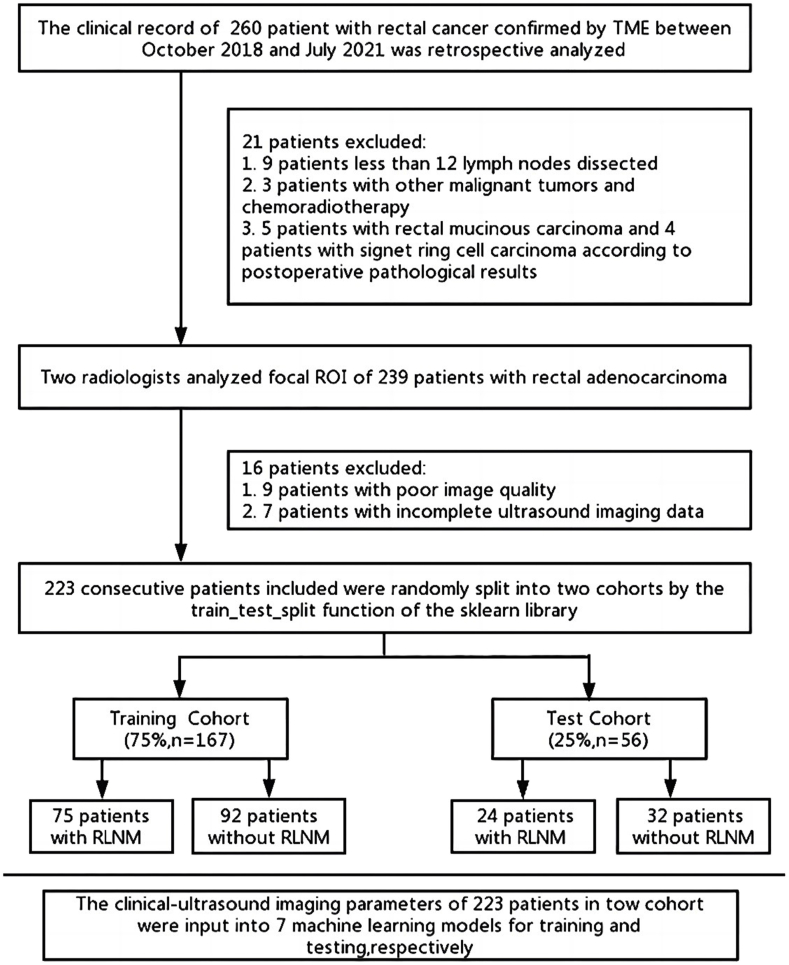
Flowchart showed inclusion and exclusion criteria of rectal cancer patients in the current study. TME, Total mesorectal excision; ROI, Region of interest; RLNM, Region lymph node metastasis.

The exclusion criteria were as follows: (1) The ultrasound image was not complete; (2) The patient suffered from other malignant tumors; (3) The patient had contraindication for SonoVue. The detailed patient recruitment pathway with inclusion and exclusion criteria is described in Fig. 1. Finally, A total of 223 patients were selected and randomly assigned by the train_test_split function of sklearn to the training cohort and test cohort at a ratio of 3:1.

This study was carried out in accordance with the Declaration of Helsinki of 1975, revised in 2008. Ethical approval was obtained from the medical ethics committees of our hospital. All patients signed informed consent and all patient details were de-identified.

### 2.2 TRUS, CEUS examinations and data analysis

**Fig. 2:**
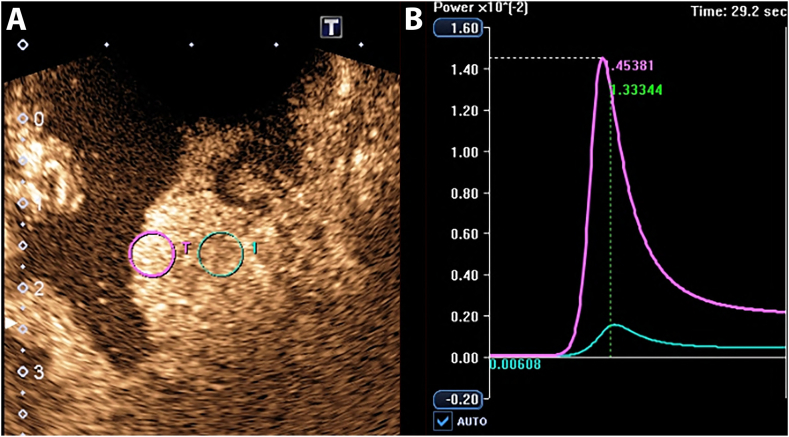
Methods of placing the region of interest (ROI) in the rectal lesion and acquiring the time-intensity curve (TIC) of the contrast-enhanced ultrasound (CEUS) image in a 65-year-old male diagnosed with moderately differentiated rectal adenocarcinoma preoperatively. After total mesorectal resection (TME), pathology showed that 0/17 lymph nodes had no cancer metastasis. (**A)** CEUS imaging at peak phase. Two ROIs were placed in the rectal cancer lesion: the strongest enhanced ROI (purple) and the weakest enhanced ROI (green). **(B)** TICs was generated from two ROIs with time on the x-axis and signal intensity on the y-axis. Use the analysis software that comes with the system, and perform fitting calculations to obtain the corresponding quantitative parameters. (For interpretation of the references to color in this figure legend, the reader is referred to the Web version of this article.)

All patients underwent TRUS and CEUS examinations within two weeks prior to TME, and pathological results were acquired. Ultrasound examinations were performed and analyzed with a Canon Aplio 500 scanner using a transrectal head-scanning probe working at a frequency of 5–10 MHz and mechanical index of 0.06–0.08. Sulfur hexafluoride (SF6) lipid-coated microbubble contrast agent SonoVue™ (Bracco SpA, Milan, Italy) was used for CEUS, which was preprocessed with 5 ml 0.9% normal saline (NS) and fully mixed before being injected. During the procedure, the patient was helped to stay in a Sims’ position and clean the rectum using enemas. The probe was slowly operated to pass through the rectum. The location, size, internal echo, and blood flow of the tumor were evaluated in various sections. The distance from the anal verge, length, thickness, peak systolic flow velocity (PSV), end-diastolic velocity (EDV), resistance index (RI), and pulsatility index (PI) of the tumor were measured. After routine TRUS examination, a section with maximum tumor size was selected, and equipment was switched to CEUS mode. A well-prepared contrast agent was administered through the forearm vein in bolus with 2.4 ml SonoVue™ within 2 s, and the following flush of 5 ml 0.9% NS was injected. Once the contrast agent was administered, the recording timer was started, and observation lasted approximately 90 s (Fig. 2a).

After CEUS, the dynamic image was stored, and the TIC was plotted using built-in software on an Aplio 500 (Fig. 2b). The equal-area method was used with a diameter of 5 mm to evaluate two ROIs, namely, the area of maximum contrast and minimum contrast. The following absolute and relative parameters were measured or calculated: peak intensity, time-to-peak (TTP), mean transit time (MTT), slope (S), area under the curve (AUC), ratio of peak intensity (PI ratio), difference of peak intensity (PI difference), difference of mean transit time (MTT difference), difference of time-to-peak (TTP difference), ratio of area under the curve (AUC ratio), and ratio of slope (S ratio). The aim of calculating relative parameters was to eliminate the impacts of other confounding factors, such as the mass of the patient, the speed of contrast agent injection, and the heterogeneity of the tumor. Two experienced radiologists who were blinded to all clinical and pathological information were unified trained to draw the region of interest (ROI) and analyze the CEUS data. Each of the ROIs was measured three times, and the average value was calculated.

### 2.3 Clinical and pathological data collection

The clinical characteristics included age, sex, preoperative carcinoembryonic antigen (CEA) (reference value ≤ 5.2 ng/ml), carbohydrate antigen (CA199) (reference value ≤ 35U/ml), and postoperative pathological lymph node status of each patient were collected.

### 2.4 Statistical analysis

The data were analyzed by SPSS 23.0 (IBM, Armonk, New York), with quantitative data described by the mean ± standard deviation and qualitative data described by N (%). The distribution of clinical and ultrasonic characteristics in both the training and test sets was compared by the Kolmogorov–Smirnov test (K–S test). A p < 0.05 was considered statistically significant. The seaborn library of Python language was used to better visualize the correlation between each parameter. The sklearn library was used to establish a machine learning model. Seven common machine learning algorithms, including Bayesian network (Bayes), K-nearest neighbor (KNN), multilayer perceptron (MLP), logistic regression (LR), support vector machine (SVM), random forest (RF), and light gradient boosting machine (LightGBM) were used to establish the prediction model, with 23 ultrasound characteristics and 4 clinical characteristics taken as independent variables and postoperative pathological lymph node metastasis as the dependent variable.

RF and LightGBM are ensemble methods that combine plural base estimators to obtain better generalization ability than a single estimator. The prediction result of RF depends on the average decision tree to avoid overfitting and obtain higher accuracy. LightGBM creates base estimators in order and makes predictions by weighted voting to obtain better performance [9]. LR is a statistical analysis that is widely applied in risk prediction [10]. SVM is a technique that uses classification algorithms to solve two-group classification problems, which is of excellent efficiency in high-dimensional space. This method is a good choice for small samples and is widely used in medical diagnosis [11]. MLP belongs to the deep neural network and has multineural layers that are brilliant when dealing with complex data [12]. Bayes is a machine learning method based on probability theory, of which the algorithm is efficient and straightforward but requires independent variables and sometimes could lose some precision [13]. KNN is a machine learning technique and algorithm based on training on the distance to find the nearest K point, which is fit for simple data.

During the training procedure, 10-fold cross validation was used to select the best hyperparameter, and the test cohort was used to evaluate the predictive performance. The model that had the best predictive performance was selected by the area under the receiver operating characteristic curve (AUC), and the importance feature was ranked according to the specific model. In addition, MedCalc software was used to process the DeLong method, and the pROC package (R language) was used to process the comparison of AUCs to evaluate the predictive performance of the seven classifiers. A *P < 0.05* was considered statistically significant. The following parameters were used to evaluate the diagnostic efficiency.

Accuracy (Acc) = (TP + TN)/(TP + FN + FP + TN).

Precision (Pre) = TP/(TP + FP).

Sensitivity (Sen) = Recall = TP/(TP + FN).

Specificity (Spe) = TN/(FP + TN).

F1-Score = 2 × Accuracy * Recall/(Accuracy + Recall).

TP (True Positive): Number of true positive cases.

TN (True Negative): Number of true negative cases.

FP(False Positive)：Number of false positive cases.

FN(False Negative): Number of false negative cases.

## 3 Result

### 3.1 Patient demographics and clinical ultrasound image features

A total of 223 patients who underwent TRUS and CEUS examinations preoperatively were enrolled in this retrospective analysis. The average age was 58.6 ± 10.8 (29–83), the ratio of males to females was 1.65:1, and the positive rate of RLNM was 43.5%. In the training cohort and test cohort, the average ages were 58.6 ± 10.7 (29–83) and 60.5 ± 10.2 (34–83), the ratios of males to females were 1.24:1 and 1.83:1, and the positive rates of RLNM were 44.91% and 42.85%, respectively. According to the K–S test, Table 1 shows that baseline clinical and ultrasonic characteristics for the two cohorts were not statistically significant (*P > 0.05*).

**Table 1:** Comparison of clinical-ultrasound image characteristics in training and test cohort.

Variables	Training cohort Mean ± SD/N (%)	Test cohort Mean ± SD/N (%)	*P* value
Number of patients	(N = 167)	(N = 56)	
Age (years)	58.6 ± 10.7 (29–83)	60.5 ± 10.2 (34–83)	1
Gender			0.376
Male	108 (64.7%)	31 (55.4%)	
Female	59 (35.3%)	25 (44.6%)	
CEA (ng/ml)			0.998
≥5.2	63 (37.7%)	26 (46.4%)	
<5.2	104 (62.3%)	30 (53.6%)	
CA199 (u/ml)			1
≥35	28 (16.8%)	8 (14.3%)	
<35	139 (83.2%0	48 (85.7%)	
Location (cm)	7.60 ± 3.32 (2–15)	6.91 ± 3.55 (2–14)	0.428
Length (cm)	4.11 ± 1.37 (0.9–8.8)	4.24 ± 1.65 (1.8–8.8)	0.987
Thickness (cm)	1.53 ± 0.62 (0.6–4.8)	1.56 ± 0.88 (0.7–5.5)	0.306
PSV(cm/s)	20.4 ± 12.1 (5.4–72.3)	19.43 ± 8.69 (7–47.9)	0.246
DEV (cm/s)	7.65 ± 5.38 (1.3–28.6)	7.11 ± 4.31 (1.6–18.5)	0.764
RI	0.60 ± 0.13 (0.11–1.21)	0.62 ± 0.13 (0.32–0.9)	0.399
PI	1.06 ± 0.31 (0.20–2.08)	1.13 ± 0.38 (0.44–2.56)	0.512
PI-max (10E-5 AU)	130.8 ± 151.6 (2.6–1422.7)	151.86 ± 130.35 (5.4–514.4)	0.656
TTP-max(s)	9.39 ± 19.2 (1.3–194.7)	7.67 ± 4.40 (2.4–26.6)	0.866
MTT-max(s)	24.9 ± 37.3 (0.9–165.1)	23.92 ± 37.22 (1.7–159.2)	0.422
Slope-max (10E-5 AU/s)	26.16 ± 32.12 (0.3–250)	30.58 ± 32.56 (0.1–140.2)	0.485
AUC-max (10E-5 AU.s)	5783.0 ± 6130.9 (58–38404.4)	7.67 ± 4.40 (193.2–222220.2)	0.623
PI-min (10E-5 AU)	39.6 ± 51.3 (0.3–345.2)	46.28 ± 53.57 (2.2–349.6)	0.356
TTP-min(s)	10.3 ± 18.5 (0.5–170.3)	7.63 ± 3.79 (2.7–22.9)	0.498
MTT-min(s)	50.9 ± 57.4 (0.8–167.8)	48.64 ± 64.16 (4.6–330.8)	0.649
Slope-min (10E-5 AU/s)	7.74 ± 10.31 (0.1–82.8)	8.73 ± 13.91 (0.5–83)	0.208
AUCmin(10E-5 AU.s)	2384.2 ± 3504.8 (3.8–26760.2)	2563.58 ± 2777.49 (115.3–14813.4)	0.330
PImax/PImin	4.33 ± 2.87 (0.17–17)	3.94 ± 2.11 (1.12–9.04)	0.222
PImax-PImin (10E-5 AU)	91.25 ± 120.03 (-286.1-1084.3)	105.58 ± 103.09 (2.5–423.4)	0.474
TTPmax-TTPmin(s)	−0.87 ± 25.72 (-150.1-194.1)	0.03 ± 5.12 (-11.8-23.9)	0.676
MTTmax-MTTmin(s)	25.9 ± 62.5 (159.4–150.1)	−24.71 ± 72.33 (-311.1-154.6)	0.465
AUCmax/AUCmin	3.62 ± 4.28 (0.37–41.41)	4.30 ± 5.98 (0.73–33.18)	0.746
Smax/Smin	5.06 ± 4.72 (0.02–31.29)	4.34 ± 4.40 (0.01–15.9)	0.883
Lymph node status			0.293
RLNM (+)	75 (44.9%)	24 (42.9%)	
RLNM (−)	92 (55.1%)	32 (57.1%)	

SD, standard deviation; CEA, carcinoembryonic antigen; CA199, carbohydrate antigen; PSV, peak systolic velocity; EDV, peak diastolic flow rate; RI, resistance index; PI, pulsatility index; PI-max/PI-min, teak intensity; TTP, time to peak; MTT, mean transit time; AUC, area under thecurve; S, slope; RLNM, region lymphnode metastasis.

### 3.2 Correlation of the RLNM status, clinical features and ultrasound parameters

**Fig. 3:**
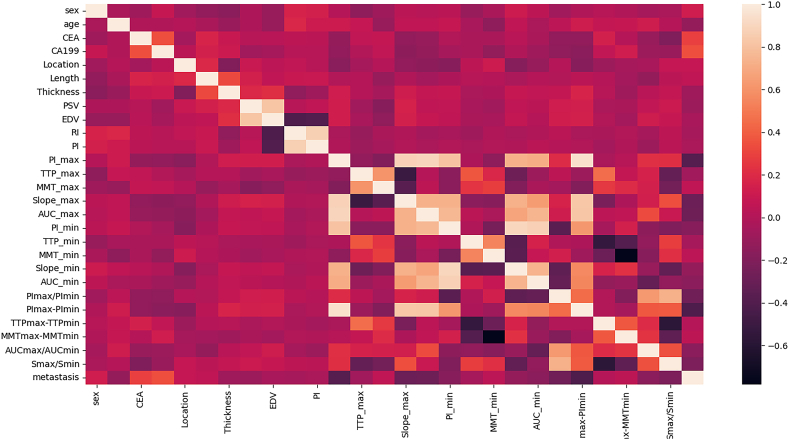
Heat-map based on the correlation coefficient between clinical-ultrasound imaging features and lymph node metastasis.

Spearman rank correlation analysis was used to evaluate the relationships of the parameters, of which correlation coefficients were visualized by a heatmap (Fig. 3). For qualitative data, CEA and CA199 correlated positively with RLNM, while age had a negative correlation. For quantitative data, PSV, PI/AUC/slope and their relative parameters had negative correlations with *RLNM*, while EDV, RI, PI, MTT/TTP and their relative parameters had positive correlations.

### 3.3 Diagnostic performance of the seven machine learning algorithms

**Fig. 4:**
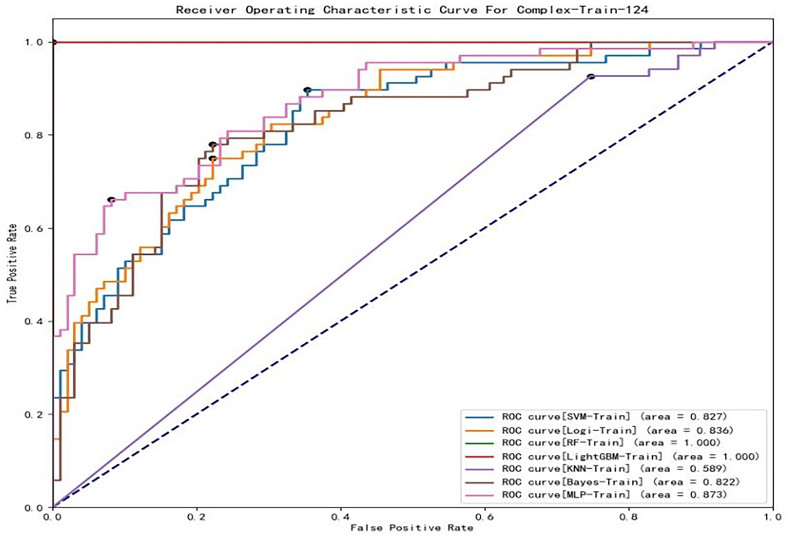
ROC curve of each machine learning model in the training cohort.

Based on the characteristics listed in Table 1, seven machine learning models were established to predict the *RLNM* of rectal carcinoma. In the training cohort. The ROCs of these models are shown in Fig. 4. The AUC of KNN, Bayes, MLP, LR, SVM, RF and LightGBM was 0.583 (95% CI:0.504–0.659), 0.822 (95% CI:0.756–0.877), 0.873 (95% CI:0.812–0.919), 0.829 (95% CI:0.764–0.883), 0.827 (95% CI:0.761–0.881), 1.000 (95% CI:0.978–1.000) and 1.000 (95% CI:0.978–1.000) (Table 2), respectively. According to the DeLong test (Table 3), the LightGBM and RF models yielded the highest AUCs, but the difference was not statistically significant (*p* = 1). The AUC of the LightGBM and RF models was better than that of SVM, LR, Bayes, KNN and MLP with statistical significance (*P* < 0.001). The AUCs of LR, Bayes (*p* = 0.732) and SVM (*p* = 0.808) were not significantly different. The KNN model had the poorest performance, and its difference from any other model was statistically significant (*P* < 0.001).

**Table 2:** Predictive performance of the seven machine learning models for training cohort and test cohort.

Model	AUC	Acc	Sen	Spe	Pre	F1 score
Training						
KNN	0.583 (0.504–0.659)	0.461	0.985	0.101	0.429	0.181
Beyes	0.822 (0.756–0.877)	0.688	0.556	0.577	0.873	0.679
MLP	0.873 (0.812–0.919)	0.802	0.676	0.889	0.807	0.842
LR	0.829 (0.764–0.883)	0.743	0.544	0.879	0.755	0.632
SVM	0.827 (0.761–0.881)	0.593	0.000	1.000	0.593	0.744
RF	1.000 (0.978–1.000)	1.000	1.000	1.000	1.000	1.000
LightGBM	1.000 (0.978–1.000)	1.000	1.000	1.000	1.000	1.000
Test						
KNN	0.517 (0.380–0.653)	0.553	0.935	0.080	0.557	0.698
Beyes	0.778 (0.647–0.878)	0.732	0.839	0.600	0.722	0.667
MLP	0.852 (0.731–0.932)	0.750	0.613	0.920	0.905	0.766
LR	0.875 (0.759–0.948)	0.679	0.483	0.920	0.882	0.719
SVM	0.867 (0.750–0.943)	0.446	0.000	1.000	0.446	0.617
RF	0.879 (0.765–0.951)	0.786	0.645	0.960	0.952	0.800
LightGBM	0.941 (0.843–0.986)	0.857	0.806	0.920	0.926	0.852

Data in parentheses are 95% CI.

AUC, area under the curve; Acc, accuracy; Sen, sensitivity; Spe, specificity; Pre, precision; Bayes, Bayesian network; KNN, K-nearest neighbor; MLP, multilayer perceptron; LR, Logistc regression analysis; SVM, support vector machine; RF, Random Forest; LightGBM, Light Gradient Boosting Machine; CI, confidence interval.

**Table 3:** Comparison between classifiers in training cohort and test cohort.

between classifiers	Training cohort		Test cohort	
	95%CI	*P value*	95%CI	*P value*
LightGBM-KNN	0.356–0.478	<0.0001	0.307–0.540	<0.0001
LightGBM-Beyes	0.113–0.242	<0.0001	0.049–0.276	0.0051
LightGBM-MLP	0.074–0.181	<0.0001	−0.008-0.186	0.0716
LightGBM-LR	0.109–0.232	<0.0001	−0.022-0.153	0.1398
LightGBM-SVM	0.110–0.236	<0.0001	−0.029-0.176	0.1597
LightGBM-RF	0.000–0.000	1.0000	−0.009-0.131	0.0863
RF-KNN	0.356–0.478	<0.0001	0.236–0.488	<0.0001
RF-Beyes	0.113–0.242	<0.0001	−0.000-0.203	0.0510
RF-MLP	0.074–0.181	<0.0001	−0.056-0.111	0.5140
RF-LR	0.109–0.232	<0.0001	−0.086-0.095	0.9224
RF-SVM	0.110–0.236	<0.0001	−0.089-0.114	0.8132
LR-KNN	0.169–0.324	<0.0001	0.216–0.499	<0.0001
LR-Beyes	−0.033-0.047	0.7320	0.000–0.193	0.0488
LR-MLP	0.015–0.071	0.0020	−0.022-0.068	0.3099
LR-SVM	−0.019-0.024	0.8080	−0.031-0.046	0.6947
SVM-KNN	0.166–0.322	<0.0001	0.199–0.500	<0.0001
SVM-Beyes	−0.044-0.053	0.8160	−0.025-0.203	0.1272
SVM-MLP	0.014–0.078	0.0050	−0.039-0.070	0.5794
MLP-KNN	0.218–0.361	<0.0001	0.194–0.474	<0.0001
MLP-Beyes	0.074–0.181	<0.0001	−0.030-0.177	0.1619
KNN-Beyes	0.156–0.323	<0.0001	0.098–0.424	0.0017

Data in parentheses are 95% CI.

Bayes, Bayesian network; KNN, K-nearest neighbor; MLP, multilayer perceptron; LR, Logistc regression analysis; SVM, support vector machine; RF, Random Forest; LightGBM, Light Gradient Boosting Machine; CI, confidence interval.

**Fig. 5:**
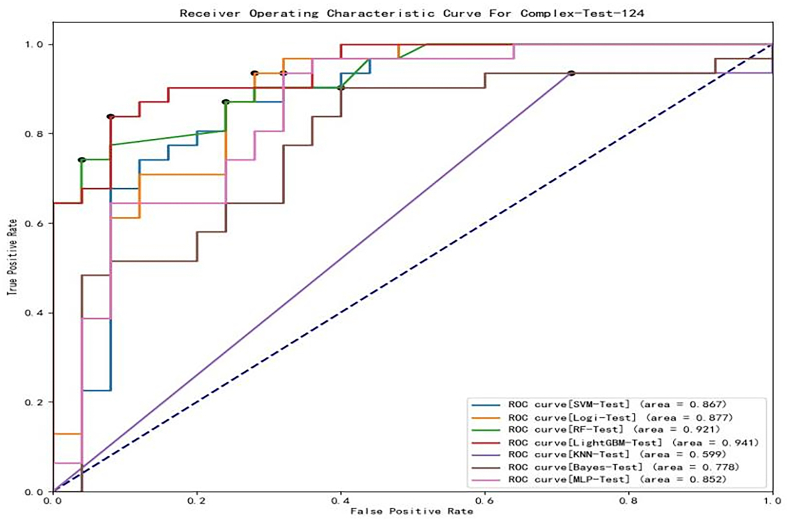
ROC curve of each machine learning model in the test cohort.

Based on the training results, these models were evaluated in the test cohort. The ROCs of these models are shown in Fig. 5. The AUC of KNN, Bayes, MLP, LR, SVM, RF and LightGBM was 0.517 (95% CI:0.380–0.653), 0.778 (95% CI:0.647–0.878), 0.852 (95% CI:0.731–0.932), 0.875 (95% CI:0.759–0.948), 0.867 (95% CI:0.750–0.943), 0.879 (95% CI:0.765–0.951) and 0.941 (95% CI:0.843–0.986) (Table 2), respectively. The difference between the AUCs of the models was evaluated and is shown in Table 3. The LightGBM model exhibited the highest AUC value among all these predictive models but did not differ significantly from SVM (*p = 0.1597*), LR (*p = 0.1398)*, RF (*p = 0.0863*) and MLP (*p = 0.0716*), while its performance was still significantly better than KNN (*p < 0.001*) and Bayes (*p < 0.05*). KNN had poor performance, and its AUC was significantly different from all other models (*p < 0.05*).

Furthermore, we used accuracy, sensitivity, specificity, precision and F1 score to compare the performances of these seven machine learning models with respect to the dataset (Table 2). In the training cohort, RF and LightGBM demonstrated the best performance based on each indicator. In the test cohort, the LightGBM model had the best AUC (0.941), accuracy (0.857), and F1-score (0.852). The SVM model had the highest specificity (1.0), the RF model had the highest precision (0.952), and the Bayes model had the highest sensitivity (0.935). The Bayes, SVM and RF models had extreme values in some evaluation indicators. Thus, the LightGBM model achieved the best and most stable performance among the seven machine learning models. From Table 2, a conclusion could be drawn that the specificity of the LightGBM model was better than its sensitivity. It has been shown that the LightGBM model can be more accurate when predicting the negative outcome of RLNM.

### 3.4 Feature importance analysis

**Fig. 6:**
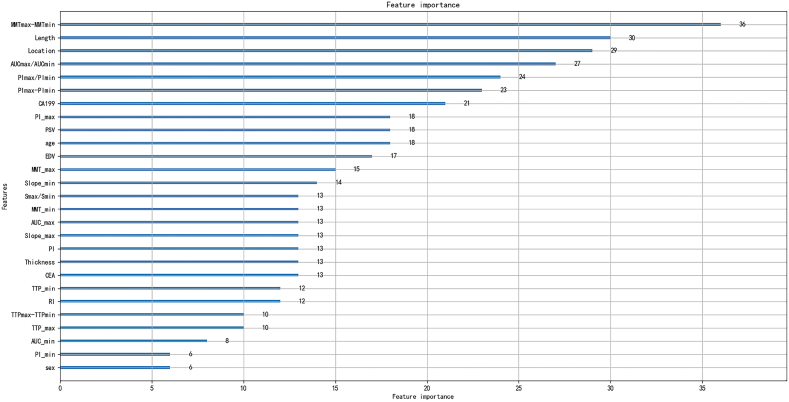
Ranking of the importance of each feature in the LightGBM model.

As previously illustrated, the LightGBM model achieved the best performance, offering a powerful tool for forecasting rectal carcinoma RLNM. The identification of feature importance based on LightGBM is shown in Fig. 6. The diagnostic performance of the LightGBM algorithm relied mainly on the following five characteristics: MTT difference (36), length (30), location (29), AUC ratio (27), and PI ratio (23). Regarding clinical characteristics, CA199 and CEA showed the most important weight. For TRUS, length and location showed the most important weight, and for CEUS, MTT difference, AUC ratio and PI ratio showed the most important weight.

## 4 Discussion

In this study, seven machine learning models were developed based on clinical ultrasound imaging to predict rectal cancer RLNM. There were three significant findings. First, these seven machine learning models could distinguish whether patients with rectal cancer had RLNM. Second, comparing the diagnostic performance between the models, LightGBM had the best predictive ability and was better at predicting negative RLNM lesions. Ultimately, the five most important factors affecting the diagnostic performance of LightGBM were the MMT difference, length, location, AUC ratio, and PI ratio.

In recent years, machine learning has been widely used in predicting tumor lymph node metastasis. Many studies [14,15] used deep learning models to predict lymph node metastasis in thyroid cancer, breast cancer, colon cancer, and gastric cancer, with AUC values as high as 0.881. Most of the studies were conducted by applying imaging histology to outline the region of interest. Ma et al. [16] predicted rectal cancer RLNM based on MRI imaging histology and found that the random forest (RF) model had the best predictive efficacy (sensitivity, 79.3%; specificity, 72.2%). Chen et al. [17]constructed an imaging histology classifier based on clinical indicators, endoluminal ultrasound and CT, which was able to predict preoperative rectal cancer RLNM more accurately, with a concordance index of 0.88. Imaging histology relies on the establishment of a large number of image databases, and there is no unified standardized research methodology at present, which is only seen in the relevant domestic and foreign literatures, and has not yet been widely used in the clinical field. However, the application of machine learning models combined with TRUS imaging parameters to predict RLNM has not yet been reported. Li et al. [18] exploited rectal cancer RLNM based on MRI imaging with the least absolute shrinkage and selection operator (LASSO) algorithm but did not compare the performance of multiple machine learning models in distinguishing RLNM in rectal cancer patients.

By training seven popular machine learning classifiers and selecting the most suitable classifier for distinguishing rectal cancer RLNM, we found that the LightGBM algorithm performed best. Compared with KNN, Bayes, SVM, LR and MLP, the AUC value of LightGBM increased by 36.5%, 17.7%, 7.9%, 5.7% and 9.5%, respectively. LightGBM was able to resist overfitting and could shorten the time to process substantial data features greatly [19], so it had an excellent generalization ability to predict rectal cancer RLNM. This study applied the optimal parameters of each model. To improve the algorithm's accuracy, tenfold cross validation was utilized to randomly divide all data into ten copies, selecting 10% of the data as the test set and the remaining 90% as the training set for verification.

The metastasis of malignant tumors is strictly related to tumor neoangiogenesis. Microvessel density is a critical indicator for predicting lymph node metastasis and distant metastasis of malignant tumors [20]. CEUS is a pure blood pool imaging method. By drawing the TIC in the ROI, the blood perfusion level in the microcirculation of the lesion was quantitatively evaluated [21]. This study found that the perfusion intensity-related parameter PI/AUC was negatively correlated with lymph node metastasis. The cause may be that most of the pathological types of rectal cancer were adenocarcinoma, and the tumor was prone to necrosis [22]. High levels of angiogenesis are often accompanied by highly aggressive tumors; in this situation, tissue hypoxia levels are higher, which could lead to easy necrosis in the tumors. Therefore, the pattern of CEUS tends to be low or have no enhancement, which means that the PI/AUC will be lower [23]. Neoangiogenesis of the tumor has the characteristics of immature development, fragile vascular wall structure, and disordered vascular distribution [24]. Wang et al. found that the more aggressive rectal cancer was, the deeper the intestinal wall infiltration and the higher the RI, which suggested the slowness of blood flow velocity. This study found that for tumors with lymph node metastasis, the blood flow rate-related parameter TTP/MTT had an increasing trend.

In this study, TIC raw quantitative analysis parameters were included and relative parameters were set when analyzing ultrasonographic perfusion information of rectal cancer lesions. This is because the raw absolute parameters can be affected by inter-patient differences and ultrasonography conditions and many other factors, such as contrast injection site, injection speed, patient body mass index, rectal lesion location, size, deviation of components, and gain, all of which affect contrast [25].The correlation parameter can effectively eliminate the interference of various factors on the data and has better robustness than the absolute parameter, because all the influencing factors have almost the same effect on the strongest and weakest enhancement areas within the lesion itself. In a study by Naoko et al. [26] on the prediction of early lymph node metastasis in breast cancer by ultrasonography, the PI ratio was found to be a more robust parameter for distinguishing the lymph node status of breast cancer compared with the original signal intensity PImin. In terms of clinical features, length and location were related to lymph node metastasis. We speculated that this was related to the accumulation of lymph nodes in the rectal area in the upper rectum, so upper rectal tumors were more prone to lymph node metastasis. The larger the tumor volume was, the longer the involved length, the deeper the invasion, and the wider the area in contact with the peri-intestinal lymphatic vessels, the greater the risk of metastasis [27].

The study has the following advantages over previous conventional imaging methods for predicting lymph node metastasis in rectal cancer. Firstly, Transrectal ultrasonography has been widely used because of its excellent characteristics such as economy, convenience and absence of radiation; Secondly, this study verified the feasibility of ultrasonography parameters and machine learning algorithms to construct a model to predict lymph node metastasis in rectal cancer. The diagnostic performance of the models is also compared and confirmed to have superior diagnostic efficacy. Finally, the ultrasonography parameters are more accessible than the mainstream imaging histology studies, while the present study adds the dimension of broadening the features (clinical features, relative and absolute parameters) and tries to validate them in the dataset, which is less mentioned in domestic and international studies, and is somewhat innovative.

To the best of our knowledge, this was the first study using multiple machine learning classifiers to diagnose rectal cancer lymph node status by using ultrasound imaging. However, there were three limitations in this study. First, it was a retrospective study with potential selection bias. We look forward to prospective clinical studies with large samples from multiple centers. Second, the region's area of interest was drawn by two different radiologists. There were specific differences in location, although the two radiologists had rich experience in ultrasound diagnosis of rectal cancer. To address this issue, in the future, it is expected that automatic segmentation techniques will be performed to automate measurements and improve clinical applicability and dissemination. Third, this study only discusses the lymph node metastasis status, and the stage of metastasis and vascular and nerve invasion need to be determined and incorporated into our new prediction model. Finally, external verification is needed to verify the accuracy and reliability of the machine learning model in the future. Further perspective studies with a larger patient would be beneficial in confirming our findings.

In conclusion, these results of this study suggested that machine learning models based on TRUS combined with CEUS, especially the LightGBM model yielded satisfactory performance in predicting the RLNM prior to surgery. These findings may provide new reference for clinical decision-making in rectal cancer.

## Ethics approval statement

This study was carried out in accordance with the Declaration of Helsinki of 1975, revised in 2008. Ethical approval (no.2023-E128-01) was obtained from the medical ethics committees of The First Affiliated Hospital of Guangxi Medical University. Informed consent was obtained from all individual participants included in the study.

## Funding

This work was supported by the Natural Science Foundation from the Technology Department of Guangxi Province, China (No. 2015GXNSFAA139172).

## Data availability statement

The data that support the findings of this study are available on request from the corresponding author.

## CRediT authorship contribution statement

**Xuanzhang Huang:** Writing – original draft, Methodology, Formal analysis, Conceptualization. **Zhendong Yang:** Writing – review & editing, Writing – original draft, Software, Formal analysis. **Wanyue Qin:** Methodology, Investigation, Data curation. **Xigui Li:** Project administration, Methodology, Data curation. **Shitao Su:** Investigation, Data curation. **Jianyuan Huang:** Writing – review & editing, Project administration, Methodology, Funding acquisition, Conceptualization.

## Declaration of competing interest

The authors declare that they have no known competing financial interests or personal relationships that could have appeared to influence the work reported in this paper.
